# Extraction of apex beat waveform from acoustic pulse wave by sound sensing system using stochastic resonance

**DOI:** 10.1038/s41598-021-92983-6

**Published:** 2021-07-01

**Authors:** Etsunori Fujita, Masahiro Horikawa, Yoshika Nobuhiro, Shinichiro Maeda, Shigeyuki Kojima, Yumi Ogura, Kohji Murata, Tomohiko Kisaka, Kazushi Taoda, Shigehiko Kaneko, Masao Yoshizumi

**Affiliations:** 1 Department of Cardiovascular Physiology and Medicine, Graduate School of Biomedical and Health Sciences, Hiroshima University, 1-2-3 Kasumi, Hiroshima, 734-8553 Japan; 2 Delta Tooling Co., Ltd, 1-2-10 Yanoshinmachi, Hiroshima, 736-0084 Japan; 3 Graduate School of Nursing, Sanyo Gakuen University, 1-14-1 Hirai, Okayama, 703-8501 Japan; 4 Office of Research and Academia-Government-Community Collaboration, Hiroshima University, 1-2-3 Kasumi, Hiroshima, 734-8553 Japan; 5 Biwako Professional University of Rehabilitation, 967 Kitasakacho, Shiga, 527-0145 Japan; 6 Major in Mechanical Engineering, School of Creative Science and Engineering, Center for Science and Engineering, Waseda University, 3-4-1 Okubo, Tokyo, 169-8555 Japan

**Keywords:** Cardiovascular biology, Circulation, Cardiology, Biomedical engineering, Physical examination

## Abstract

With a sound sensing system using stochastic resonance (4SR), it became possible to obtain an acoustic pulse wave (APW)—a waveform created via a mixture of apex beat and heart sound. We examined 50 subjects who were healthy, with no underlying cardiovascular diseases. We could determine boundary frequency (BF) using APW and phonocardiogram signals. APW data was divided into two bands, one from 0.5 Hz to BF, and a second one from BF to 50 Hz. This permitted the extraction of cardiac apex beat (CAB) and cardiac acoustic sound (CAS), respectively. BF could be expressed by a quadratic function of heart rate, and made it possible to collect CAB and CAS in real time. According to heart rate variability analysis, the fluctuation was 1/f, which indicated an efficient cardiac movement when heart rate was 70 to 80/min. In the frequency band between 0.5 Hz and BF, CAB readings collected from the precordial region resembled apex cardiogram data. The waveforms were classified into five types. Therefore, the new 4SR sensing system can be used as a physical diagnostic tool to obtain biological pulse wave data non-invasively and repeatedly over a long period, and it shows promise for broader applications, including AI analysis.

## Introduction

Visual inspection and forms of palpation and auscultation are used as basic diagnostic methods^1^, which occupy important positions in physical examination in cardiology. A phonocardiogram (PCG) delivers an objective graphing of the auscultation method, which is the visualization of vibrations and sounds derived from the heart and great arteries in the 16 to 1000 Hz audible range. On the other hand, apex cardiogram (ACG), the target vibration frequency range of which is below 10 Hz, corresponds with visual inspection and palpation^2^. However, the ACG sensor’s major drawback is its susceptibility to noise^3^, since piezoelectric elements are commonly employed. Among previous approaches regarding signal processing to extract information from ACG, the Stretchable E-Skin Apexcardiogram Sensor^4^ can analyze waveforms and time phase of the A wave, but it was reported that recording skills might largely affect waveforms other than that of the A wave. And gyrocardiography^5^ has not been able to reproduce the ACG waveform. Consequently, both methods are mainly used for time phase analysis. No machine learning method conducting automatic detection of the ACG waveform has been reported so far.

In 2015, we completed a sound sensing system using resonance (3SR^6,7^) with a mechanical amplification mechanism using a harmonic oscillator.

In 2019, we developed a new sound sensing system using stochastic resonance (4SR) to measure human vibration waveforms below 10 Hz. 4SR combines a harmonic oscillator at 20 Hz with a stochastic resonance mechanism^8^. We succeeded in extracting the acoustic vibration information of 0.5 to 80 Hz from the apex by using this system.

Since the previous study^9^ reported that there was a frequency around 16 Hz which was the boundary between apex beat and heart sound, we decided to further examine the correlation between heart rate and BF (which is a boundary between CAB and CAS) by using heart rate variability (HRV) and statistical methods, focusing on BF.

The 3SR system was destined to collect biological information from the back, and we named this information an aortic pulse wave (APW), considering that it mainly captured HRV from heart sound via the aorta. The 4SR system was developed to extract apex beat waves, with the aim of measuring acoustic signals from the precordial, back and lumbar regions. Therefore, in this paper, we named the biological information collected by 4SR an acoustic pulse wave, although it uses the same basic APW abbreviation. To avoid confusion, we distinguish these APWs by adding a prefix: APW measured from the precordial region as front acoustic pulse wave (F-APW), for instance.

## Results

### Performance of the 4SR sensing system and measurement of F-APW

**Figure 1:**
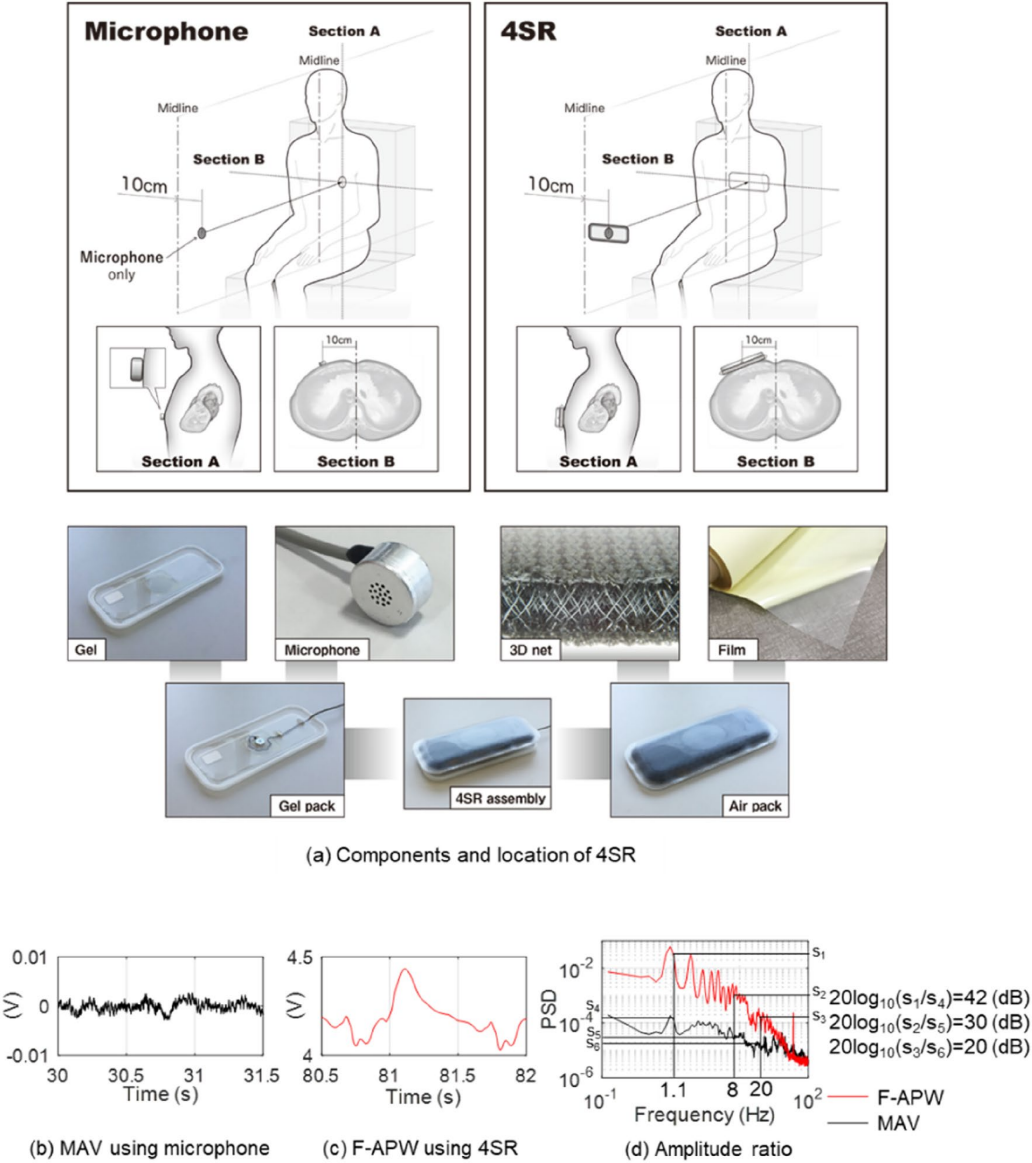
Experimental apparatus and characteristics of 4SR (amplitude ratio). (**a**) The installation position and component configuration of 4SR. 4SR is an assembly comprising of a gel-pack (left photo) and an air-pack (right photo). The gel-pack consists of gel injected into a soft plastic case and a microphone. The air-pack is sealed with an elastomer film and embedded three-dimensional knitted fabrics called 3D net (see the sectional photo) of 10 mm thickness. The air-pack and the gel-pack are fixed together by adhesion of the gel. For comparison, micro acoustic vibration (MAV) was collected using only the microphone. (**b**) MAV obtained using only the microphone. (**c**) F-APW obtained using 4SR. (**d**) The amplitude of apex beats and heart sounds increased almost two 100-fold, and the gain was 20 to 42 dB through mechanical vibration system response and the stochastic resonance effect.

Figure 1a shows the installation position of the sensor and the component configuration of 4SR to evaluate its sensing performance. 4SR consists of a gel-pack and an air-pack. The gel-pack consists of gel injected into a soft plastic case and a microphone. The air-pack is sealed with an elastomer film and consists of three-dimensional knitted fabrics known as 3D net (see the sectional photo in Fig. 1a) of 10 mm thickness. It is conceivable that 4SR amplifies vibrations of 0.5 to 80 Hz by the two resonance mechanisms of stochastic resonance and string vibration. Thus, the elastomer film produces tension by receiving a uniform air pressure, delivering conditions which make it easy to propagate acoustic vibration.

Micro acoustic vibration (MAV) emitted from the body trunk enables the microphone in the gel-pack to detect and measure a signal of a certain frequency band caused by the frictional vibration of 3D net in the air-pack.

During testing, a microphone and 4SR were placed 10 cm left of the chest midline on the left fifth intercostal space.

Figure 1b shows MAV being measured by the microphone. Since the microphone is attached directly to the chest, MAV information is unaffected by the amplification effect of stochastic resonance. Figure 1c shows F-APW measured by 4SR. F-APW is acoustic vibration information which is affected by the responses of the mechanical vibration system comprising the 4SR sensing system, harmonic oscillator resonance and stochastic resonance. Then, Fig. 1d shows the amplitude ratios calculated by frequency analysis of the time waveforms of MAV and F-APW.

The gains of F-APW/MAV were 42 dB in a band of the fundamental vibration of apex beats, slightly more than 30 dB in a band of higher harmonics, and 20 dB in the vicinity of 20 to 30 Hz, which is the band of the harmonic oscillator and a minimum band of heart sounds. The effect of the response of the mechanical vibration system and stochastic resonance indicated that the maximum gain was over 40 dB and the minimum was 10 dB, while its amplitude difference increased by slightly less than 200 times.

**Figure 2:**
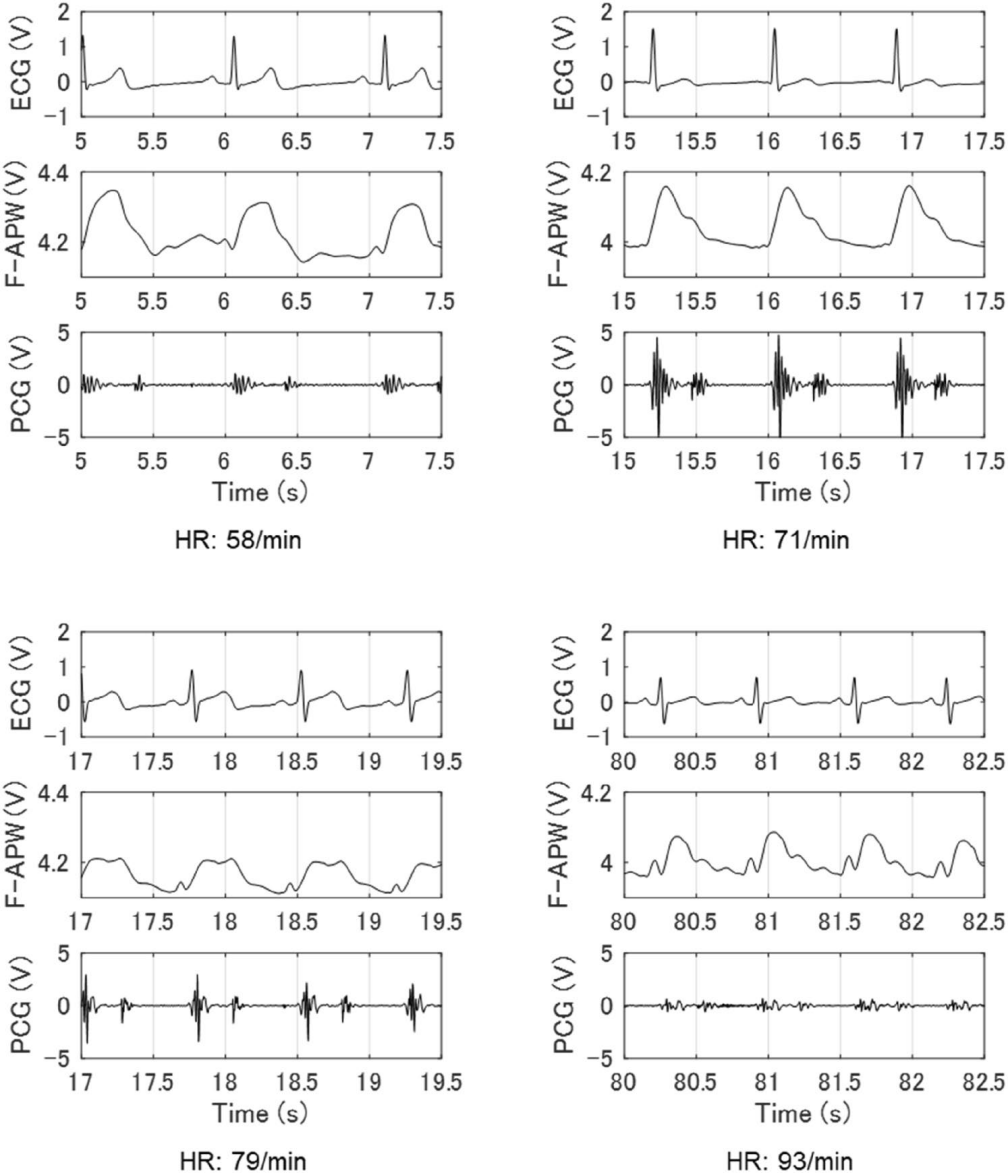
ECG, F-APW and PCG by different heart rates. F-APW measured from the precordial region, ECG and PCG readings of subjects in a sitting position whose mean heart rates for 10 s are 58, 71, 79 and 93/min, respectively.

Figure 2 shows the time waveforms for 2.5 s of electrocardiogram (ECG), F-APW and PCG of four subjects, whose average heart rates were 58, 71, 79 and 93/min, respectively. Waveforms in a 1 to 1.5 Hz band were observed in F-APW.

### Determination of BF

**Figure 3:**
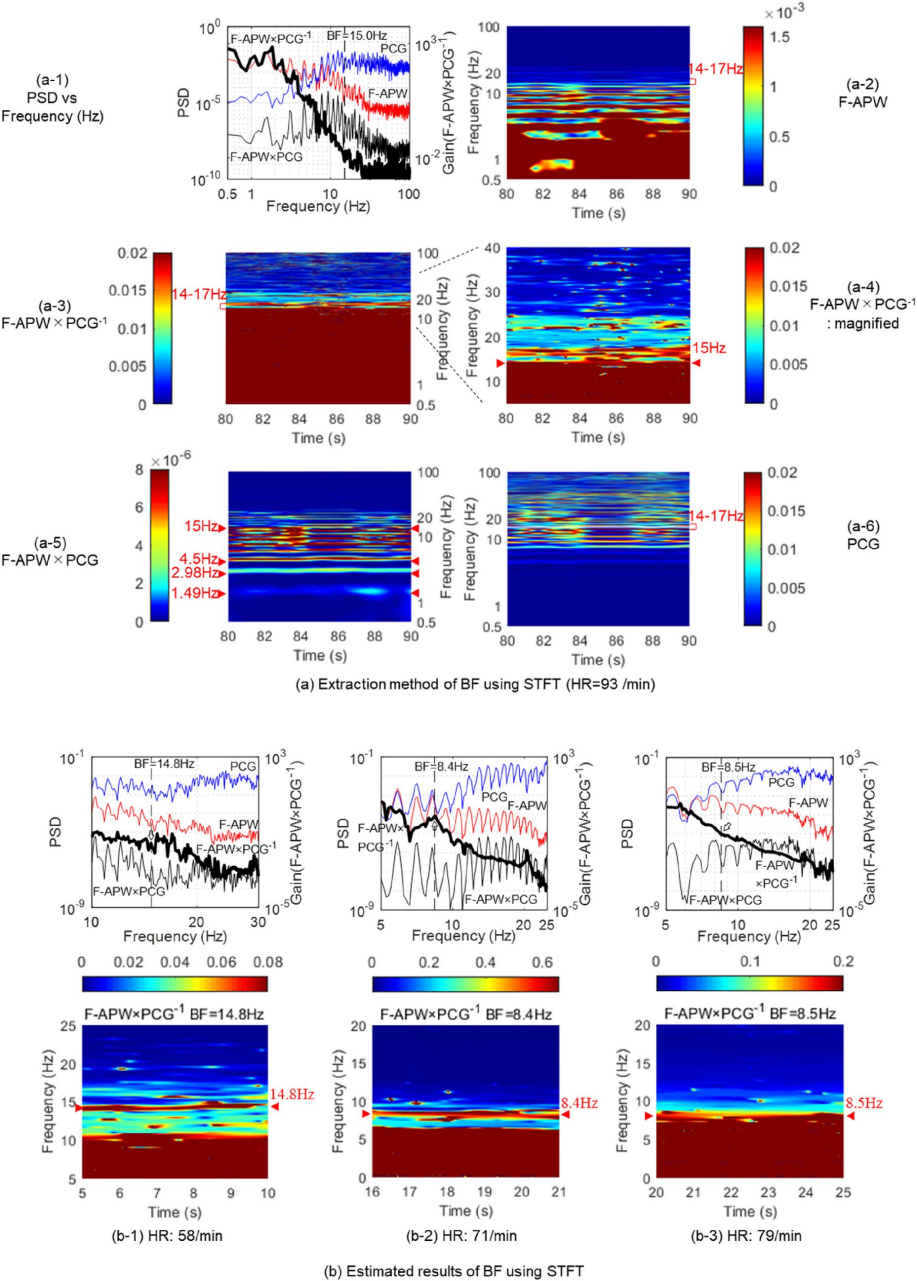
Determination of BF using STFT. The results of frequency analysis and STFT analysis of the same subjects (heart rates are 58, 71, 79 and 93/min) in Fig. 2. (**a-1**) The power spectra of F-APW × PCG^−1^, F-APW, PCG and F-APW × PCG of the subject whose mean heart rate is 93/min. The X axis is frequency, while the Y axis is power spectral density (PSD). The breakpoint is in the vicinity of 15.0 Hz of F-APW × PCG^−1^. (**a-2**) STFT analysis of F-APW. F-APW is comprised of higher harmonics up to ninth order, the fundamental vibration of which is 1.6 Hz. (**a-3**) STFT analysis of F-APW × PCG^−1^. (**a-4**) A magnified figure of a-3. The Red line in the vicinity of 15 Hz is defined as Boundary Frequency (BF). (**a-5**) STFT analysis of F-APW × PCG. (**a-6**) STFT analysis of PCG. (**b**) The results of frequency analysis and STFT analysis of F-APW × PCG^−1^ of the other three subjects (heart rates are 58, 71 and 79/min). The arrow heads indicate BF.

**Figure 4:**
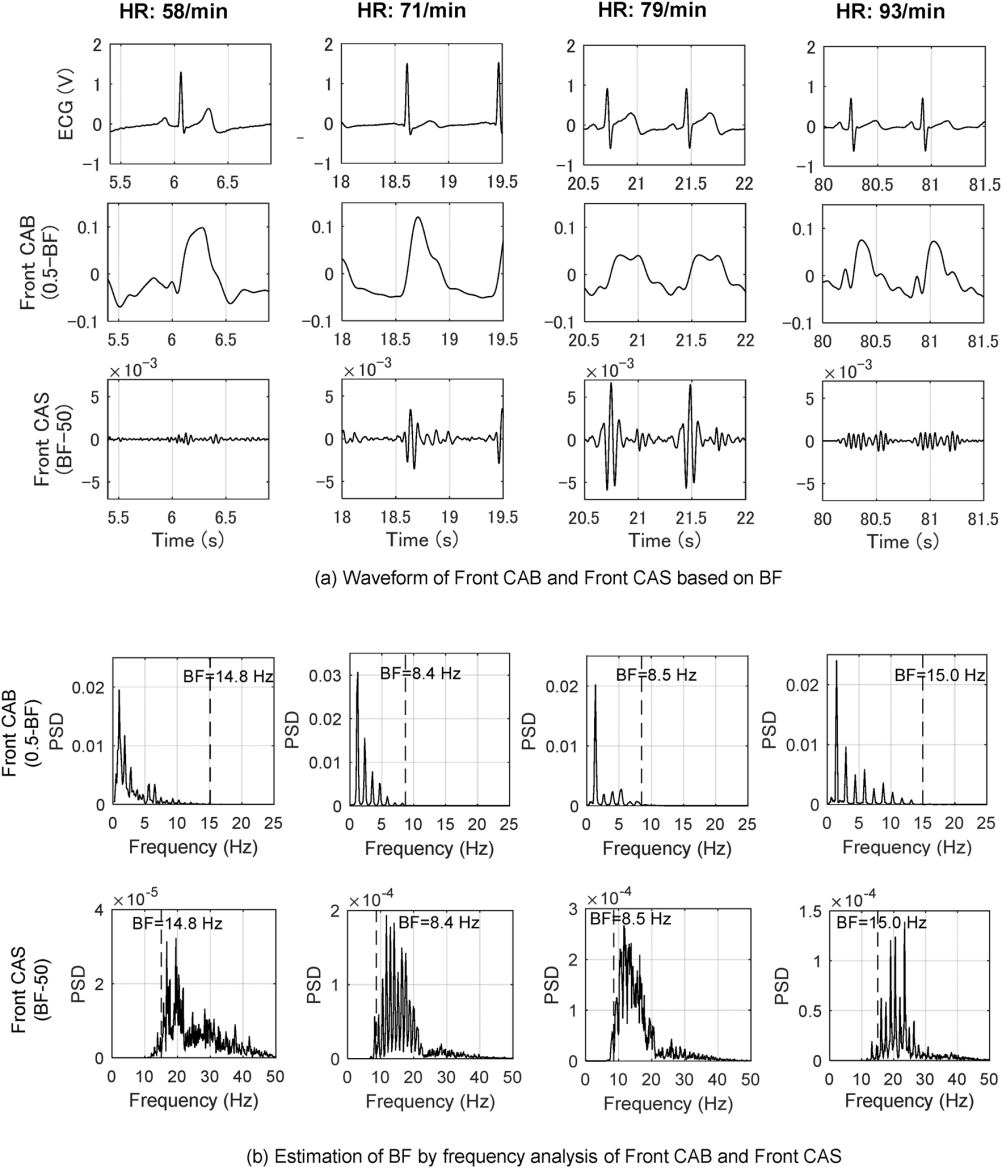
Extraction of CAB and CAS from F-APW and confirmation of BF. **(a)** According to Fig. 3, BF of the same subjects as Fig. 2 (heart rates are 58, 71, 79 and 93/min) were 14.8, 8.4, 8.5 and 15.0 Hz, respectively. CAB in the range of 0.5 Hz to BF and CAS in the range of BF to 50 Hz were extracted from F-APW collected from the precordial region based on BF. Front CAB (0.5-BF) resembles apex beat wave, and CAS resembles PCG. **(b)** Frequency analysis was conducted of Front CAB and Front CAS obtained from F-APW of the four same subjects based on BF determined in Fig. 3. It was revealed that the higher harmonic components of Front CAB decreased and converged in the vicinity of each BF.

**Figure 5:**
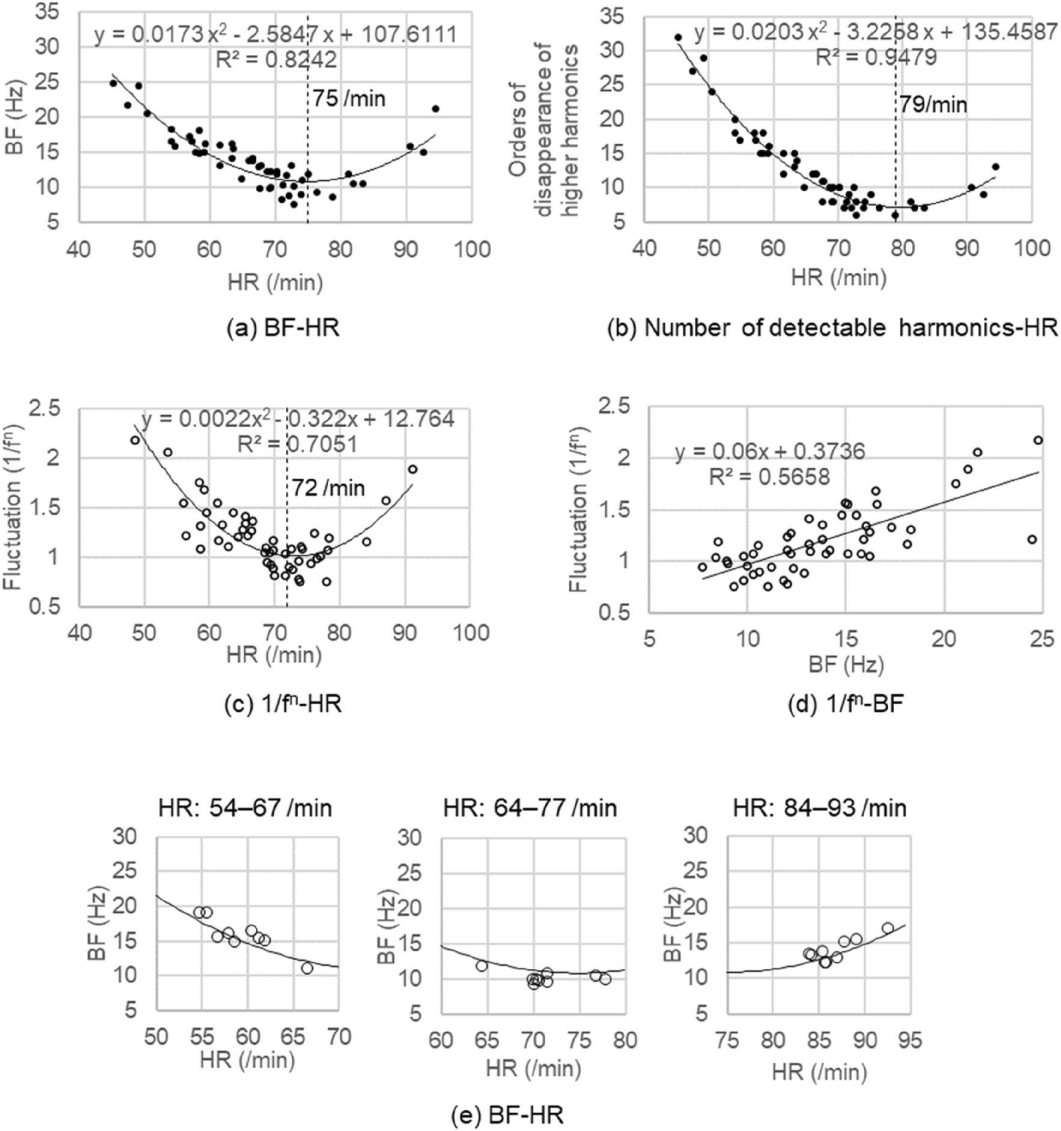
Correlation diagram of heart rates, BF, orders of disappearance of higher harmonic vibration, and 1/f^n^. (**a**) Correlation diagram of BF-HR, (**b**) number of detectable harmonics-HR and (**c**) 1/f^n^-HR were represented by a quadratic function.(**d**) 1/f^n^-BF was approximated by a linear function. The fluctuation became 1/f (|n|= 1) when BF was in the vicinity of 8 to 12 Hz and heart rate was around 72/min. On the other hand, the fluctuation was 1/f^2^ (|n|= 2) when BF was more than 20 Hz and heart rate was in the vicinity of either 50 or 90/min. (**e**) When variation of heart rates of the three subjects occurred under respiratory depression at rest, BF moved along the quadratic curve of the BF-HR correlation diagram shown in **(a)**.

**Figure 6:**
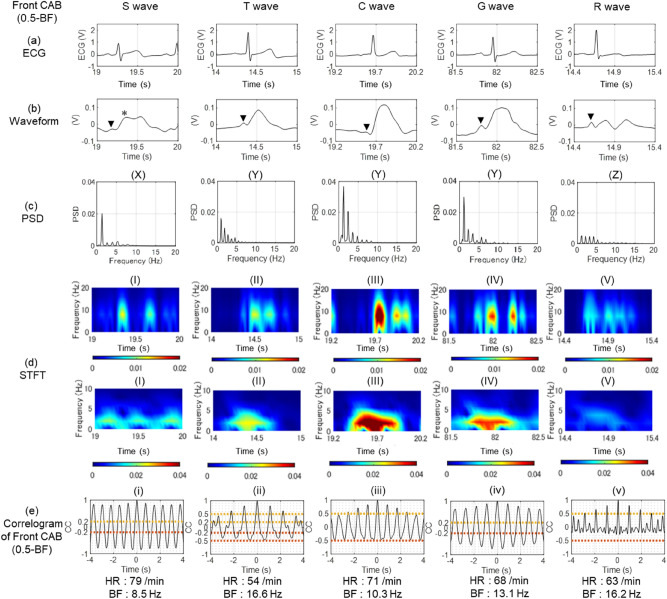
Classification of Front CAB (0.5-BF) waveform. (**a**) The time waveforms for one second of ECG of five subjects. (**b**) The waveforms of Front CAB (0.5-BF) are classified into Square (S), Triangular (T), Cosine (C), Gauss (G) and Random (R) by their forms. The “A” wave of ACG (wave of atrial contraction: arrow head) and a similar waveform of ventricular systolic wave, which starts from the E point (*), are recognized. (c) PSD obtained by the frequency analysis of Front CAB (0.5-BF) revealed power spectra of the fundamental vibration and higher harmonics. PSD was divided into three patterns: Pattern (X) in which only the fundamental vibration is predominent, pattern (Y) in which the higher harmonics declined from the fundamental vibration naturally, and pattern (Z) in which multiple higher harmonics existed without diminishing from the fundamental vibration. (d) STFT analysis of the Front CAB (0.5-BF) waveforms was performed depending on two frequencies, 1.95 Hz for a power spectrum component of the A waveform (five lower panels), and 7.81 Hz for that of the E point (five upper panels). Results of STFT analysis were classified into five patterns of (I),(II), (III), (IV) and (V). Figure 6d shows STFT figures of the Front CAB (0.5-BF) time waveforms to readily conduct pattern matching by doubling the information amount on the power spectrum with two frequencies of 1.95 Hz and 7.81 Hz. They were classified into five patterns of (I), (II), (III), (IV) and (V). (e) Of the correlograms of Front CAB (0.5-BF), (i) which is a sine wave, (ii) which is a waveform with a notch on a sine wave, and (v) which is a waveform with two or more notches on a sine wave, are extracted. The remaining waveforms are divided into (iii), in which one side of the envelope curve is decayed, and (iv), in which both sides of the envelope curve are decayed.

Figures 3a-1–a-6 show results of frequency analysis and short time Fourier transform (STFT)^10^ by the frequency analysis of F-APW and PCG of a subject whose average heart rate for 10 s was 93/min. BF corresponding to a breakpoint^11^ is 15 Hz.

Figures 3b-1–b-3 show results of frequency analysis and STFT of subjects whose average heart rates were 58, 71 and 79/min, respectively.

Figure 3a-1 shows the power spectra of F-APW × PCG^−1^, F-APW, PCG, and F-APW × PCG. F-APW × PCG^−1^ has a breakpoint at 15 Hz, which is BF.

Figure 3a-2 shows that BF in the Figure indicates a sudden changing point of the power spectrum as a breakpoint, it exists between 14 and 17 Hz, and is shown in Blue.

Figure 3a-3 shows F-APW × PCG^−1^. Since BF indicates a sudden changing point in a range in which power spectrum variation is small as a breakpoint, it exists between 14 and 17 Hz.

Figure 3a-4 shows a magnified view of F-APW × PCG^−1^, and a Red line to indicate a breakpoint that appeared in the vicinity of 15 Hz.

F-APW × PCG in Fig. 3a-5 shows a Blue line indicating the fundamental vibration of 1.49 Hz, a Green line as the second order (higher) harmonic, and a Red line as higher harmonics from the third order up to 15 Hz, which shows that the higher harmonics have a higher power spectrum than that of the fundamental vibration.

It further shows that there is a breakpoint in the Blue range, indicated by Red-Blue-Red in the vicinity of 15 Hz, and random vibration between 15 and 20 Hz, in which Red appears.

Figure 3a-6 shows that PCG has a vibrational component whose power spectrum variation is large between 10 and 50 Hz.

The higher harmonics of apex beats and variation behavior in the random vibration of heart sounds were detected by the two drawn lines of F-APW × PCG^−1^ (Fig. 3a-4) and F-APW × PCG (Fig. 3a-5), which made it possible to identify a frequency for the disappearance of higher harmonic vibration of apex beats, and a frequency for heart sound appearance and BF.

Figures 3b-1–b-3 show BF obtained via a BF extraction method using F-APW × PCG^−1^ and STFT. The heart rates of the subjects were 58, 71 and 79/min, respectively.

Figure 3b-1, in contrast with Fig. 3a-1, shows that a breakpoint appeared on a chevron-shaped power spectrum, and BF appeared on the Red line in a band between Red-Green–Red-Blue.

Figure 3b-2 shows that the breakpoint indicated by Fig. 3a-1 appeared on the chevron-shaped power spectrum, and BF appeared as a Red line between Red-Blue-Red-Blue.

Figure 3b-3 shows that fluctuation of the power spectrum varied, and BF appeared on the boundary of Red-Blue as a Red line using the STFT method of the figure below, although it is difficult to determine it from the results of frequency analysis of the figure above.

### Extraction of CAB and CAS from F-APW

Figure 4 shows ECG, apex beat (Front CAB) and heart sound (Front CAS) waveforms drawn from F-APW, and the results of frequency analysis by heart rate (58–93/min). Figure 4a shows waveforms of CAB and CAS drawn by filtering using BF, and they are shown as Front CAB (0.5-BF) and Front CAS (BF-50). The numbers and BF in parentheses show frequency bands of filtering. The ranges of measurement on the vertical and horizontal axes of the waveforms of CAB and CAS have been standardized. Figure 4b shows results of frequency analysis of Front CAB and Front CAS on linear scales by heart rate (58–93/min). This figure highlights the disappearance of the harmonic component of CAB in BF, appearance of the random vibration of CAS in the vicinity of BF, and BF’s dependence on heart rate. The proposed extraction methods of acoustic vibration information in this paper enabled the separation of CAB and CAS, and visualization of their characteristics for the first time. It is revealed that Front CAB (0.5-BF) of the four subjects are drawn as apex beat waves, which are mainly composed of the fundamental vibration. Heart sounds are random vibrations composed of high frequency waveforms that combine multiple vibration waveforms.

In addition, the BF extraction method based on the frequency of disappearance of higher harmonic vibration is an approach to determine BF by frequency, while the BF extraction method based on STFT uses a power spectrum threshold in order to determine BF. Specifically, the former method is an approach to determine the frequency using the height of the power spectrum at its bottom and peak as BF, while the latter method ascertains BF by the threshold, which is 70 percent of the average of the power spectrum group in the vicinity of BF. BF can be established using these two methods.

### Correlation diagram of BF, heart rates, and 1/f^n^ of HRV

Since preliminary experiments and examinations revealed that heart rate was involved in BF, we created a correlation diagram of BF and heart rate using data from 50 subjects in order to investigate the relationship between the two.

Figure 5a is a correlation diagram of BF-heart rate (HR) of the 50 subjects confirmed by BF extraction method from a frequency of disappearance of higher harmonic vibration and STFT. Putting BF on the vertical axis and heart rate on the horizontal axis, an approximation of their relation by a quadratic function indicates that the relation is y = 0.0173x^2^ − 2.5847x + 107.6111 which becomes the minimum value in the vicinity of 10 Hz when heart rate is 75/min. At this time, R^2^ = 0.8242. The use of the present figure enables us to know BF without measuring PCG. Figure 5a is composed of data measured for 10 s in which RR interval fluctuation is within 15 percent in a resting state.

BF had no correlation to age, systolic blood pressure (SBP), diastolic blood pressure (DBP) and body mass index (BMI). However, a further examination of BF in a control group of 20 young subjects indicated a strong correlation (R^2^ = 0.85) between BF determined by STFT and BF calculated by the regression curve of BF-HR.

Figure 5b is a correlation diagram between orders of disappearance of higher harmonic vibration and heart rate. Putting the orders on the vertical axis and heart rate on the horizontal axis, an approximation of their relation by a quadratic function indicates that the relation is y = 0.0203x^2^ − 3.2258x + 135.4587 which becomes the minimum value in the vicinity of 10 Hz when heart rate is 79/mm. At this time, R^2^ = 0.9479.

We examined the correlation between the fluctuation characteristics of HRV and heart rates obtained from the ECG of the 50 subjects in the F-APW measurement experiment. As shown in Fig. 5c, the relation between the two was approximated by a quadratic function, and when heart rate was 72/min, the fluctuation became 1/f (|n|= 1)^12,13^.

BF is determined by heart rate (for 10 s in this case). The tendency of the value of HRV fluctuation (for 90 s) obtained from BF matched that of the value of HRV fluctuation (for 90 s) obtained directly from the heart rate. The results obtained therefore support the validity of BF.

Figure 5d shows a correlation diagram between the fluctuation characteristics of HRV and BF.

Their relation was approximated by a linear funciton. The fluctuation became 1/f (|n|= 1) when BF was in the vicinity of 8 to 12 Hz and the fluctuation became 1/f^2^ (|n|= 2) when BF was in excess of 20 Hz.

Figure 5e shows the variation of BF when HRV is generated under respiratory depression at rest, achieved by dividing the experimental data results for 90 s of the same subjects into nine equal parts and described on the regression curve of the BF-HR correlation diagram. BF of the three subjects moved along the regression curve in a similar fashion to HRV.

### Classification of Front CAB time waveforms

We classified and evaluated Front CABs (0.5-BF) measured from the 50 subjects to define normal patterns in a sitting position. Figure 6a shows the time waveforms for one second of ECG of five subjects. The waveforms of Front CAB (0.5-BF) were classified into five main waveforms shown in Fig. 6b. Then, we named these waveforms square wave, triangular wave, cosine wave, Gauss wave and random vibration wave, respectively.

The square wave and Gauss wave correspond to sustained patterns which are supposed to capture a duration of a systolic wave on inspection and palpation, while the triangular wave, cosine wave and random vibration wave correspond to tapping patterns^14^.

Waveform analysis requires the waveform of the fundamental vibration and the higher harmonics, amplitude and frequency of each waveform in the vicinity of each bottom and peak, and time phase.Fig. 6c shows the results of frequency analysis of Front CAB (0.5-BF) and the results of classification of the relations between amplitude and frequency obtained by this analysis. Figure 6d shows the results of STFT analysis, which are images of the power spectra of the fundamental vibration and the higher harmonics of Fig. 6c. Figure 6d is power spectrum information divided into two parts—one is between the fundamental frequency and 5 Hz (of which the center frequency is 1.95 Hz), and the other is between 5 and 10 Hz, with its center frequency of 7.81 Hz. The information on the fundamental vibration and the higher harmonics of 1 to 5 Hz of Fig. 6c is drawn between 1 and 5 Hz of STFT. Incidentally, the information on the time waveform is expressed by the contour of Red, Green and Blue.

The waveform (I) indicates it is a square wave with three time zones in which fundamental vibration appears, and two time zones where the components of 5 to 10 Hz appear. The appearance interval of the A wave and E point is short.

The waveform (II) indicates it is a triangular wave where STFT shows the appearance of components less than 5 Hz focus at between 2.3 and 2.6 s, while components of 5 to 10 Hz concentrate between 2.5 and 2.7 s, and these components occur within the systolic phase.

Also, the waveform (III) indicates it is a cosine wave which falls within the systolic phase, and its interval between A wave and E point is short.

Though the waveform (IV) has STFT characteristics of waveforms (II) and (III), it indicates Gauss wave characteristics since its interval of peak values between 5 to 10 Hz is close to that of the waveform (I).

The waveform (V) shows a random wave which indicates that the STFT peak values of less than 5 Hz is a contour line downward to the right. The power spectrum magnitude remains the same between the fundamental vibration and the higher harmonics. Furthermore, it has more than three peak values between 5 and 10 Hz, and their appearance time phase resembles that of the components of less than 5 Hz.

We also classified vibration types via a correlogram of Fig. 6e using autocorrelation coefficients which are more than 0.5 and less than 0.2 as the standard value, and by the notch shape on sine waves.Hence, of the correlograms of Front CAB (0.5-BF), (i) is a sine wave, (ii) is a waveform with a notch on a sine wave, and (v) is a waveform with two or more notches on a sine wave. The remaining waveforms are divided into (iii), in which one side of the envelope curve is decayed, and (iv), in which both sides of the envelope curve are decayed.

### Examination of Front CAB time waveforms

Using Fig. 6b,c, we examined the waveforms of 50 examples through pattern matching. As a result, the appearance ratios of the square wave, triangular wave, cosine wave, Gauss wave and random vibration wave were 4/50 = 8%, 8/50 = 16%, 15/50 = 30%, 11/50 = 22% and 12/50 = 24%, respectively. Table 1 shows the result of waveform examination using two types of STFT from Fig. 6c, the power spectrum from Fig. 6d and correlogram from Fig. 6e, and without the waveform examination defined by Fig. 6b. The red figures in Table 1 indicate the number of examinations in which waveforms were determined. Of the 50 examples in total, 43 were able to be determined by STFT and correlogram. As for the power spectra, it was possible to determine 38 of the 50 examples. The P-value was 2.2 × 10^–16^ by STFT examination, 7.9 × 10^–5^ by power spectrum examination, and 2.2 × 10^–16^ by correlogram examination. In accordance with the Wiener-Khintchine theorem, the STFT and correlogram have a relation of inverse Fourier transform, so that the correspondence of the P-values of both confirms the validity of the analysis method. Consequently, performing these three examinations together suggests the following possibilities:

**Table 1:**
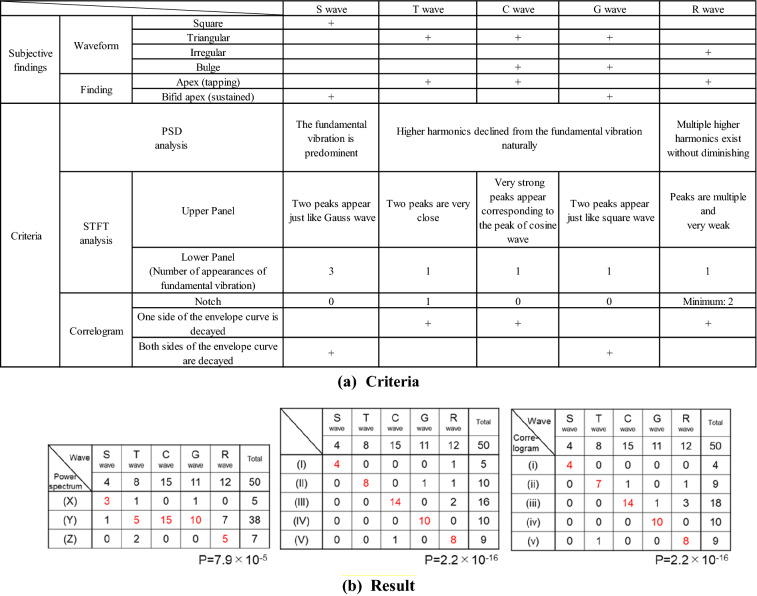
Classification of Front CAB (0.5-BF) waveform.

(a) The upper half of the Criteria table indicates the classification points by visual inspection of Front CAB (0.5-BF). We conducted analysis of each waveform, using PSD (power spectral density) when focusing on amplitude, STFT on amplitude and phase, and a correlogram to check if they have periodicity. We classified the waveforms in accordance with the criteria described in the lower part of the table.

(b) This shows that the visual classification of the waveforms of Front CAB (0.5-BF) corresponds quite well to classification by PSD analysis, STFT and correlogram.

The possibility of improving the precision of waveform examination of Front CAB (0.5-BF).

The possibility of classifying ACG waveforms^15^ recorded so far with a high level of precision.

## Discussion

The correlation diagram between BF and heart rate was made using a combination of two BF extraction methods, one of which was based on the frequency of disappearance of higher harmonic vibration, and the other using STFT. The actual measurement results indicated that BF could be expressed by a quadratic function of heart rates, enabling the determination of BF via F-APW data only and in real time, without the use of a PCG. Since BF moved along the regression curve of the correlation diagram between BF and heart rate when HRV occurred in respiratory depression during rest, the vibration analysis and the correlation diagram described in this paper suggested a possibility of movement analysis of the heart and evaluation of pressure pulsation, the condition of heart movement, and ventricular hemodynamics. Furthermore, the visualization method by STFT suggested a possibility, not only of vibration analysis of heart, but also of a diagnosis utilizing the degree of variation.

The experimental results of HRV fluctuation suggested that when the heart rate was between 70 and 80/min in a resting state and when BF was in the vicinity of 10 Hz, the fluctuation became 1/f, which indicated an efficient level of exercise.

Although BF was manually determined according to the algorithm in this paper, the finding of a BF-HR correlation enabled the acquisition of CAB and CAS via F-APW data in real time at this time. We will automate the determination of BF, and aim to fully automate all steps in the future.

We obtained the biological pulse wave (F-APW), and succeeded in its objective evaluation via the 4SR system and the analysis algorithm established in this research.This method makes it possible to collect biological information non-invasively and repeatedly over a long period, and shows promise in broader applications, which could be further enhanced as AI analysis evolves.

## Methods

### Measurement method of F-APW

The fundamental principles allowing amplitude amplification of 4SR are the harmonic oscillator and the stochastic resonance phenomenon, and both are created by the air-pack containing 3D net. The upper and lower surfaces of the 3D net are base fabric layers made of multifilament yarn, while the pile in the middle is a monofilament material interwoven with the base fabric layers, and ultimately bound through friction bonding with the multifilament yarn. A subject’s weight applied to the 3D net surface causes flexion of the X-shaped pile of the 3D net built into the air-pack, and friction on the pile caused by body movement creates a certain amount of sound and vibration. This sound and vibration causes the stochastic resonance phenomenon. Furthermore, flexing the X-shaped pile generates tension, which results in creating a harmonic oscillator. The natural frequency of the X-shaped pile under tension is in the region of 20 Hz. The air-pack, containing 3D net and air, acts as a spring and damping structure, the circumference of which is sealed with elastomer film. The gel-pack is also a spring and damping structure, which has a spring characteristic close to that of the air-pack. The air-pack and the gel-pack are fixed together by the adhesion of the gel.

As shown in Fig. 1a, first, a microphone (EM186 model, from Primo, Japan) was placed 10 cm left of the chest midline on the fifth intercostal space (equivalent to the vicinity of ECG lead V4) to measure MAV.

Next, we placed 4SR in the same position to measure F-APW, recording the results on a data logger to enable us to carry out the following analysis by filtering. We evaluated sensing performance using gain, which was the ratio of the power spectrum of 4SR to the microphone (PSD_F-APW_/PSD_MAV_) obtained by frequency analysis of input waveforms recorded on the data logger.

All measurements were carried out in a sitting position. Figure 1a shows the placement of 4SR. A microphone for PCG was placed on the apex. Also, ECG was measured by Lead II. The F-APW measurement experiment in a resting state was conducted as follows:

1. Subjects to breathe naturally for 5 min.
2. To hold their breath for 30 s after 2 s of inspiration.
3. To breathe naturally for 30 s.
4. To hold their breath for 30 s after 2 s of inspiration.

We selected 50 subjects (39 male and 11 female) aged in their 20 s to 60 s who were healthy, with no underlying cardiovascular disease.

Clinical profiles of the 50 subjects in this research are as follows: The mean age is 38.2 (± 10.3) years old, and the mean BMI is 23 (± 3.4); the group includes 4 people with hypertension and 10 smokers.

We conducted a further examination in a control group of 20 young all-male subjects to verify the data of the aforementioned 50 subjects. The clinical profiles of the 20 subjects for validation are as follows: The mean age is 23.4 (± 3.6) years old, and the mean BMI is 20.8 (± 2.4) including 1 person with hypertension and 5 smokers.

For analysis, we used 10 s of data from the initial 30 s with breath held from the part where RR interval variation was within 15 percent, and used the mean value of this data to establish heart rate.

In addition, in accordance with the Declaration of Helsinki, this study was approved by the ethics committee within Delta Kogyo Co. Ltd, and informed consent was obtained from the subjects by explaining the purpose and contents of the experiment before it was performed.

### BF extraction (higher harmonic vibration disappearance)

An apex beat wave consists of fundamental vibration and harmonic vibration comprised of higher harmonics. Hence, we conceived a method to find a changing point between higher harmonics and random vibration in a power spectrum via frequency analysis of F-APW waveforms obtained with 4SR attached to a precordial region, and averaging the spectra. The power spectrum of the CAB higher harmonic components within the apex beat wave becomes smaller with an increase of its frequency. On the other hand, the power spectrum of the random vibration system of CAS within heart sound does not depend on the frequency, but its magnitude does change.

In this paper, we refer to the frequency where the power spectrum of CAB higher harmonics decreases, and the variation behavior of CAS has been termed as BF.

**Figure 7:**
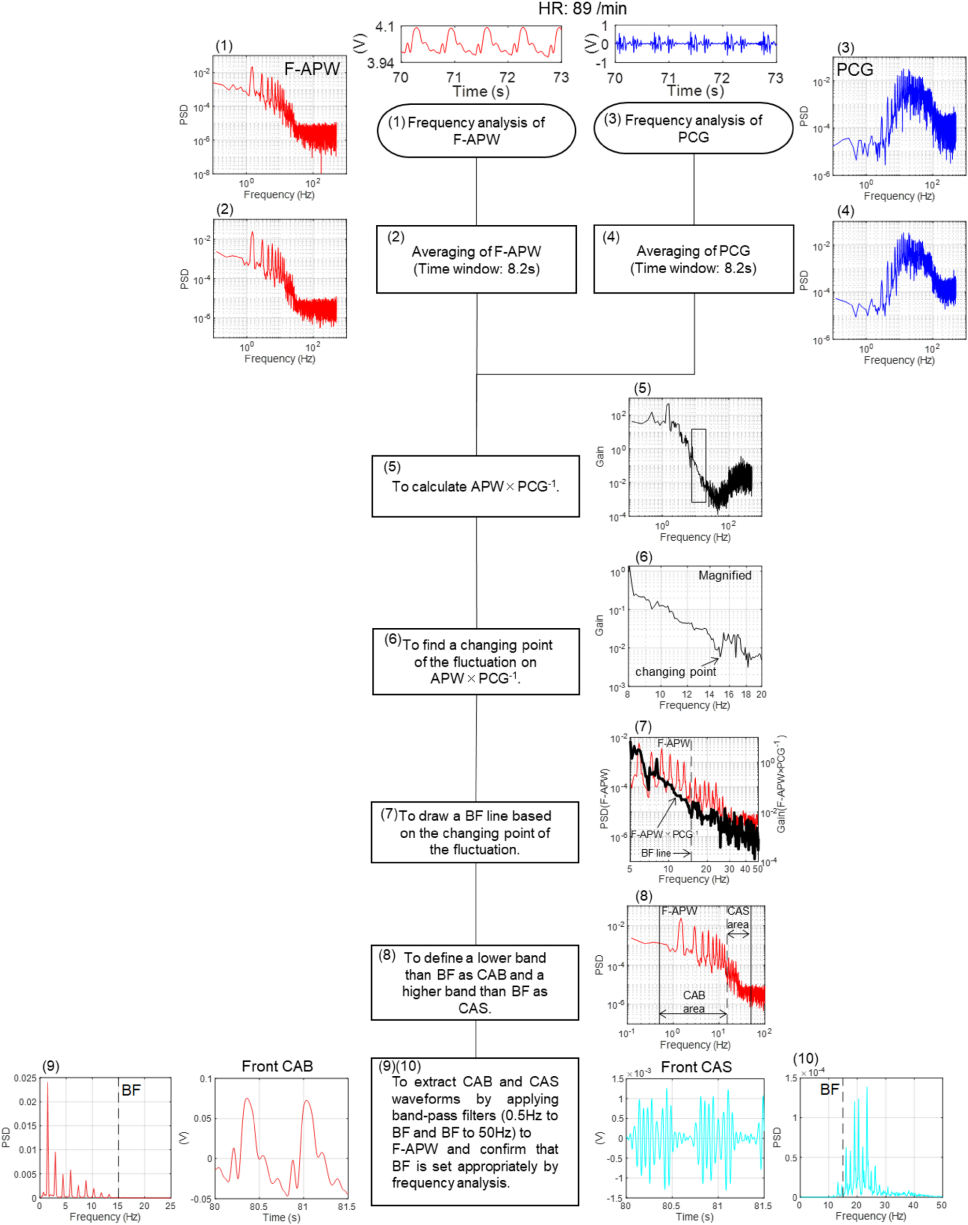
Flow chart: the way to compute BF.A flow chart to estimate BF by obtaining F-APW × PCG^−1^ from F-APW and PCG. Front CAB and Front CAS waveforms can be extracted from F-APW using the obtained BF. While F-APW × PCG^−1^ indicates the frequency analysis result of F-APW divided by the frequency analysis result of PCG, it also means that the frequency analysis waveform of PCG subtracts from the frequency analysis waveform of F-APW on a logarithmic axis. A cliff part appears in the differential waveform shown in drawing (6) of Fig. 7, and is considered to be BF.

Figure 7 shows a flow chart which shows the process for extracting BF from the power spectrum of F-APW × PCG^−1^. The following describes the calculation process and viewpoint, with the numbers labelled (1) to (10) corresponding to those shown in Fig. 7:

1. To obtain F-APW from 4SR data without filtering, and conduct frequency analysis.
2. To conduct averaging of the frequency analysis results of F-APW (the time window is 8.2 s and overlapped 90%).
3. To extract a waveform from PCG and conduct frequency analysis.
4. To conduct averaging of the frequency analysis results of PCG (the time window is 8.2 s and overlapped 90%).
5. To calculate F-APW × PCG^−1^ using the power spectra of F-APW and PCG obtained by (2) and (4) to find a breakpoint.
6. To identify the frequency of disappearance of higher harmonic vibration from the apex beat wave having fluctuation with the frequency of appearance of the random vibration component of heart sounds, which is white noise by the breakpoint on the waveform of F-APW × PCG^−1^.
7. To draw a BF line from the disappearing point of higher harmonics on F-APW × PCG^−1^ and define the intersection of the BF line with F-APW as BF.
8. To define a frequency band of 0.5 Hz to BF as the CAB band and a frequency band of BF to 50 Hz as the CAS band.
9. To apply a band-pass filter (0.5 Hz to BF) to F-APW, and confirm that the higher harmonic component of CAB is decreasing in the vicinity of BF.
10. To apply a band-pass filter (BF to 50 Hz) to F-APW, and confirm that CAS is random vibration in the vicinity of BF.

Considering that there may be cases in which it would be difficult to find BF because of the different impedance of transmission characteristics of the acoustic vibration of the trunk, due in part to the variation amongst subjects, we discovered a frequency of disappearance of higher harmonic vibration of the apex beat waveform by applying a logarithmic difference method as another means of establishing BF.

The Fourier transform of F-APW is conducted by averaging the time window of 8.2 s, and overlapping it 90 percent. Then, the waveform processed by averaging is shown on a double logarithmic chart. Incidentally, PCG is used as the heart sound element for the logarithmic difference method.

### BF extraction method using STFT

By applying STFT to F-APW, PCG, F-APW × PCG and F-APW × PCG^−1^, the changing point of fluctuation can be visualized. The equation of STFT used in this paper is as follows:

1 $${\text{STFT}}\left( {\uptau ,\upomega } \right) = \mathop \smallint \limits_{{ - \infty }}^{\infty } x\left( t \right)W\left( {t - \tau } \right)e^{{ - j\omega t}} dt$$

where *x(t)* is a measurement of signal data, and *W(t* *−* *τ)* is a window function. To make the frequency resolution 0.5 Hz or less, we select 2^12^ = 4096 as window function points. 4096 points corresponds to 4.096 s at the sampling frequency of 1000 Hz, and consequently the frequency resolution is 0.244 Hz. Also, the moving point of the window function for averaging was set to 50 points. *t* shows a time measurement and *τ* shows time series data based on the moving time of the window function. By multiplying the measurement signal data by the window function, it is possible to create frequency information which changes with time. In addition, the frequency information occurs every 0.05 s (50 divided by 1000 is 0.05 s).

In the STFT figures, the vertical axis shows frequency and the horizontal axis shows output time of STFT. The shade of color illustrates the degree of the power spectrum variation, with Red, Green and Blue (RGB) showing the magnitude of the power spectrum variation. Planar Red, Green and Blue appear in areas where the power spectrum variation is small and spectral characteristics of a signal autocorrelation function^16^ (fluctuation coefficient^17^) do not change suddenly, and in random vibration with a small amplitude. On the other hand, linear Red, Green and Blue appear in areas where the power spectrum variation is large and the fluctuation coefficient changes suddenly.

### Correlational analysis (BF, heart rates, 1/f^n^ of HRV)

In this paper, we refer to a power specrum gradient described on a double logarithmic chart as fluctuation coefficient, and a rapid changing point of the fluctuation coefficient as the changing point of fluctuation hereinafter. At the changing point of fluctuation, loss of complexity^18^ and a breakpoint occur.

The fluctuation coefficient is expressed by a power spectrum (*P(f)*) and a frequency (*f*) which become (*logP(f)*) and (*log(f)*) on a double logarithmic chart, and when the relation between *logP(f)* and *log(f)* is linear, *logP(f)* is expressed by the following equation:

2 $$\log P\left( f \right) = \kappa - n\log \left( f \right) = \kappa + \log \left( {1/f^{n} } \right)$$

where *κ* is the constant and *n* is the fluctuation coefficient. We use the experimental data obtained through the experimental protocol of F-APW measurement to evaluate the fluctuation characteristics of HRV obtained from ECG. Fluctuation characteristics are calculated using Eq. (2).

Since Musha et al*.* proposed to define the value of *n* as 1/f fluctuation in 1982^19^, the obtained values of *n* shall be expressed with an absolute value—a negative value of about − 1.5 to − 0.5 as it happens.

To evaluate the fluctuation of HRV, we use 90 s of data, which consists of apnea for 30 s, natural breathing for 30 s and apnea for 30 s. Heart rates used here are the mean value over 90 s.

### Analysis method of time waveform of Front CAB

ACG is one of the physical diagnostic tools for evaluating cardiovascular hemodynamics noninvasively and are indices of pathological conditions and treatments. During examination, the body is not only in a supine position, but also a sitting position and left lateral decubitus position in some cases. Hence, information regarding normal apex beat patterns in each body position of measurement (which covers supine position, sitting position and left lateral decubitus position) enables examination on relationships within waveform analysis and time analysis of PCG and ECG.

Then, we obtain Front CAB (0.5-BF) by extracting the frequency band of 0.5 Hz to BF of F-APW waveform, measured by the microphone attached on the chest wall and analyzed by frequency, and compare it with a clinically known apex beat waveform.

As a viewpoint of waveform analysis, we focus on the tapping pattern of the E wave, which is a short duration pulsation recognized only in the early systole on inspection and palpation, and also a sustained pattern which is a strong and long duration pulsation. In this study, we conduct waveform analysis using the waveforms of Front CAB (0.5-BF) measured in a sitting position and those in the systolic phase in which the peak of ACG appears.

The bottom value of the amplitude of the time waveform is between the P wave and R wave of ECG. The peak value of the amplitude of the time waveform is between the R wave and the starting point of the second heart sound of PCG. Next, we classify the time waveforms of Front CAB (0.5-BF) of the 50 subjects.

### Higher harmonics component analysis method using STFT

For the higher harmonics which create the time waveform of Front CAB (0.5-BF), we investigated on which part of the time waveform the component of higher harmonics was superimposed by using Eq. (1).

The frequency is divided into 1 to 4 Hz and 4 to 16 Hz for analysis. The time window points are set to 512 (2 to the ninth power) for waveform analysis in the 1 to 4 Hz range. The 512 points correspond to 0.512 s at a sampling frequency of 1000 Hz, and the 1.95 Hz power spectrum component appears according to our calculation. Setting the moving points of the Hanning window function at 51 points generates frequency information every 0.051 (= 51/1000) s.

The window points are set to 128 (2 to the seventh power) to capture an E wave comprised of the higher harmonics. 128 points correspond to 0.128 s at a sampling frequency of 1000 Hz, and a power spectrum component appears at 7.8 Hz. The reason for setting the resolution to 0.12 s (7.8 Hz) is because we determined a required frequency resolution to be 0.13 s in order to detect the peak of the duration of the systole, assuming the duration of the systole is 0.26 s when heart rate is 90/min.

In this case, setting the moving points of the Hanning window function to 12 points generates frequency information every 0.012 (= 12/1000) s.

### Vibration distinction method using autocorrelation function

We use an autocorrelation function (ACF) to draw a correlogram^20^ and sharply distinguish vibrations, such as sinusoidal vibration and random vibration.

The vertical axis of correlogram is ACF, while the horizontal axis is time. We calculate ACF at each time interval (T) and capture the waveform types and periods from the correlogram.

In addition, ACF, or ${R}_{x}\left(\tau \right)$ that is, is expressed by the following equation:

3 $$R_{x} \left( \tau \right) = \mathop {\lim }\limits_{{T \to \infty }} \frac{1}{T}\mathop \smallint \limits_{0}^{T} x\left( t \right)x\left( {t + \tau } \right)dt = \mathop {\lim }\limits_{{T \to \infty }} \frac{1}{T}\mathop \smallint \limits_{{ - \frac{T}{2}}}^{{\frac{T}{2}}} x\left( t \right)x\left( {t + \tau } \right)dt$$

where τ indicates time interval and lag, and T indicates the entire interval.

The maximum value by Normalized ACF becomes 1 when τ = 0, and its value is root mean square value. If ACF value is 0.2 or less, it exits at an interval of 95 percent or higher confidence of not having autocorrelation. Vibration type analysis is conducted using 10 s of data.

### Theoretical basis and objective criteria of waveform classification

By setting an arbitrary periodic function to be f(t), f(t) can be expanded via the following Fourier series, provided that the period shall be T and ω = 2π/T.

4 $$f\left( t \right) = a_{0} + \mathop \sum \limits_{{n = 1}}^{\infty } \left( {a_{n} \cos n\omega t + b_{n} \sin n\omega t} \right)$$

As for the S wave, since its waveform is close to that of a sine wave, it corresponds to the situation in which a first component predominates in the Eq. (4). However, as it contains some higher harmonic components, from the STFT results, it can be interpreted that the fundamental frequency is a core component accompanied by the higher harmonic components. This interpretation corresponds to the findings of a sustained pattern having a long systolic duration.

The T wave is close to a triangular wave, so that it can be expanded via the following Fourier series:

5 $$f\left( t \right) = \frac{{8V_{m} }}{{\pi ^{2} }}\mathop \sum \limits_{{n = 0}}^{\infty } \frac{{\cos \left( {2n + 1} \right)\omega t}}{{\left( {2n + 1} \right)^{2} }}$$

Equation (5) indicates that there is a characteristic that as the order number of n becomes higher, the expansion coefficient rapidly becomes smaller, and also indicates that the order appearing as a spectrum is an odd order, which can explain the form of PSD.

It is possible to explain that the autocorrelation coefficient indicated by a correlogram has its peak at t = 0, while the coefficient and its absolute value become less than 1 at other times, due to the fact that waveforms of other higher harmonics than the sine wave affect the coefficient.

This means that a waveform derived from an acute ventricular contraction having a short duration in the early systole is superimposed on the sine wave, which corresponds with the findings of the tapping pattern having a short duration in the early systole.

The same consideration as the T wave can be applied to the waveform of the C wave derived from the ventricular contraction. The main difference is that the predominance degree of the first component in the C wave is higher than that in the T wave.

In the case of Fourier series expansion of the Eq. (5), with the fundamental frequency and second higher harmonic as the results of STFT analysis being core components, a waveform derived from the ventricular contraction is formed by these frequency components up to the seventh higher harmonic. This indicates that the same correlogram as that of the T wave corresponds to the findings of the short duration tapping pattern wave in the early systole.

As for the G wave, it can be considered from STFT analysis that it is a combination of the S wave and T wave, and since the first component dominates and it is closer to the T wave than the S wave, its duration in systole is short, which corresponds to the findings of the sustained pattern.

Lastly, as for the R wave waveform derived from the ventricular contraction, if we hypothesize that its waveform has multiple peaks instead of a single peak and it resembles a full-wave rectified waveform, it can be expanded using the following Fourier series:

6 $$f\left( t \right) = V_{m} \left[ {\frac{2}{\pi } - \frac{4}{\pi }\mathop \sum \limits_{{n = 1}}^{\infty } \frac{{\cos 2n\omega t}}{{4n^{2} - 1}}} \right]$$

It is conceivable that the Eq. (6) begins with the second component and is followed by even order higher harmonics superimposed on it. As n becomes bigger, the expansion coefficient becomes smaller. Incidentally, it is estimated that the R wave has the form indicated in the figure of PSD, since it also contains the first component.

From the analysis of the correlogram, it is possible to understand the reason why the components other than the first one predominate as well as the T wave, which corresponds to the findings of the tapping wave having a short duration in the early systole.

## Abbreviations

3SR: Sound sensing system using resonance
4SR: Sound sensing system using stochastic resonance
ACF: Autocorrelation function
ACG: Apex cardiogram
APW: Aortic pulse wave (biological pulse wave collected by 3SR) → acoustic pulse wave (biological pulse wave collected by 4SR)
BMI: Body mass index
BF: Boundary frequency
CAB: Cardiac apex beat
CAS: Cardiac acoustic sound
DBP: Diastolic blood pressure
ECG: Electrocardiogram
F-APW: APW measured from the front (precordium) region
F-APW × PCG: Power spectrum—frequency diagram which emphasizes the PCG component of F-APW by addition on double logarithmic axes (multiplication on linear axes)
F-APW × PCG^−1^: Power spectrum—frequency diagram which decreases the PCG component of F-APW by subtraction on double logarithmic axes (division on linear axes)
Front CAB: Cardiac apex beat waveform drawn from F-APW
Front CAS: Cardiac acoustic sound waveform drawn from F-APW
HR: Heart rate
HRV: Heart rate variability
MAV: Micro acoustic vibration
PCG: Phonocardiogram
SBP: Systolic blood pressure
STFT: Short time Fourier transform
